# Breastfeeding Practices and Associated Factors in Shanghai: A Cross-Sectional Study

**DOI:** 10.3390/nu14204429

**Published:** 2022-10-21

**Authors:** Yanhui Hao, Lulu Wang, Caifeng Wang, Aiping Peng, Wei Gao, Isabelle Marc, Sonia Semenic, William D. Fraser, Anuradha Narayan, Yanting Wu, Suying Chang, Hefeng Huang

**Affiliations:** 1 Obstetrics and Gynecology Hospital, Institute of Reproduction and Development, Fudan University, Shanghai 200032, China; 2 School of Nursing, Shanghai Jiao Tong University, Shanghai 200025, China; 3 The International Peace Maternity and Child Health Hospital, School of Medicine, Shanghai Jiao Tong University, Shanghai 200030, China; 4 Department of Pediatrics, Research Center of CHU de Québec-Université Laval, Faculty of Medicine, Laval University, Québec, QC G1V 0A6, Canada; 5 Ingram School of Nursing, McGill University, Montréal, QC H3A 0G4, Canada; 6 Department of Obstetrics and Gynecology, Université de Sherbrooke, Sherbrooke, QC J1K 2R1, Canada; 7 United Nations Children’s Fund, Office for China, Beijing 100600, China; 8 Research Units of Embryo Original Diseases, Chinese Academy of Medical Sciences (No. 2019RU056), Shanghai 200011, China

**Keywords:** cross-sectional survey, exclusive breastfeeding, infants and young children, maternal knowledge, breast milk, breast-milk substitutes marketing

## Abstract

The status of breastfeeding practices remains unsatisfactory across China, but regional differences persist. However, disaggregated data for specific provinces are limited. This representative survey determined the status of breastfeeding and factors associated with breastfeeding practices in Shanghai. The questionnaire was designed in compliance with indicators for assessing infant and young child-feeding practices defined by the World Health Organization and the United Nations Children’s Fund (UNICEF). A total of 2665 children aged two years and younger (0–730 days) were investigated, among whom 1677 were aged under six months. The early initiation of breastfeeding (EIBF) rate was 60.3%. Among children aged under six months, 43.4% were exclusively breastfed (EBF). The univariate regression analysis showed that the EBF rate was influenced by multiple factors, including individual, socioeconomic, workplace and employment, and health system. The subsequent multivariate analysis suggested that mothers with a higher rate of EBF shared the following characteristics: intention to breastfeed during pregnancy, breastfeeding knowledge, and higher satisfaction with support through the healthcare system after delivery. The rate of EBF in Shanghai is over 40%, and supporting breastfeeding requires measures at multiple levels, including individual attributes, women’s work and employment conditions, breastfeeding knowledge, and health services.

## 1. Introduction

Breastfeeding plays an irreplaceable role in child survival, nutrition, and physical and mental development. There is a wealth of evidence suggesting that children are protected from diarrhea and respiratory infections in the short term and benefit through higher intelligence and probable lower risks of obesity and diabetes in the long term [1,2]. For nursing women, breastfeeding protects against postpartum depression, breast cancer, ovarian cancer, diabetes, and even nonalcoholic fatty liver diseases [3,4,5]. The World Health Organization (WHO) recommends that breastfeeding should be initiated within the first hour after birth and that infants should exclusively breastfeed for the first 6 months; complementary foods should then be introduced, with continued breastfeeding until 24 months of age or older [6]. However, the aggressive marketing of breast-milk substitutes (BMSs) has led to a sales boom: according to the 2019 edition of the UNICEF report *The State of the World’s Children*, sales of milk-based formula grew by 41% globally and by 72% in upper/middle-income countries such as Brazil, China, and Turkey from 2008 to 2013, while only two in five infants under six months of age are exclusively breastfed worldwide [7,8]. In China, the exclusive breastfeeding rate is even lower than the global average.

The breastfeeding rates conducted in different periods and different cities and areas in China have shown diversity [9]. In 2013, a national representative survey (Chinese National Nutrition and Health Survey) conducted across 55 counties in 30 provinces indicated that only 20.7% of the 14,539 children were exclusively breastfed in their first six months. The survey also found that the exclusive breastfeeding rate in urban metropolises was 18.9%, slightly higher than the 18.5% found in general rural areas [10,11]. In 2018, a cross-sectional survey conducted by Shi et al., including 5237 infants from 31 provinces, reported that 29.5% of infants under six months were exclusively breastfed [12]. Another study published in 2020, with 9745 mother–child dyads from seven regions in China, reported that the rate of EBF was 27.8%, and the prevalence of exclusive breastfeeding under 6 months was lower in medium and small cities, as well as rural areas, when compared with big cities [13]. Shanghai, the largest megacity in China, has a relatively high per capita income, a higher proportion of the population with a higher educational level, and advanced maternal and child healthcare services. The characteristics of breastfeeding practices in Shanghai may therefore differ from those in the countryside.

A systematic review performed by *The Lancet* Breastfeeding Series Group included a conceptual model with the components of an enabling environment for breastfeeding, including sociocultural context, health-system practices and support, the impact of BMS marketing, the experience and attitudes of family members, employment status, breastfeeding intentions, and confidence at the individual level [14]. It is necessary to investigate current breastfeeding practices in Shanghai and identify the obstacles and favorable factors toward increasing the rate of exclusive breastfeeding in the first six months following birth up to at least 50% by 2025.

## 2. Methods

### 2.1. Study Design and Study Participants

A cross-sectional survey was carried out in the childcare clinics of 36 community hospitals in the Xuhui, Changning, Minhang, and Fengxian districts in Shanghai, with the support and guidance of the UNICEF China Office. The inclusion/exclusion criteria of the participants were in line with the Sino-Canadian Healthy Life Trajectories Initiative (SCHeLTI) [15]. Inclusion criteria included a maternal age between 20 and 42 years old, and the youngest child being of a singleton birth and aged under two. Women were excluded if they had received in vitro fertilization (IVF) treatment; had been diagnosed with HIV, hepatitis B/C, severe heart disease, or autoimmune disease; or their child had a severe congenital malformation. From May 2020 to January 2021, 3004 mothers were approached at community hospitals, and 2665 questionnaires were completed and included in the final analysis. Of these, 1677 children were aged 0–5 months and 988 aged 6–23 months. The response rate was 88.7%, which was higher than we expected when calculating the sample sizes (85%). The major reason why respondents did not answer questions in surveys was that some respondents were not comfortable with providing personal information.

Ethical approval was obtained from the International Peace Maternity and Child Health Hospital (IPMCH) Ethics Committee in Shanghai (GKLW2017-01). All surveyed mothers provided digital informed consent before they completed the online questionnaires at the community hospitals. Strict confidentiality was ensured by all research staff members. Personal information or any data of the participants remained anonymous throughout the study.

### 2.2. Questionnaire, Data Collection and Management

A standard questionnaire for mothers was developed based on the WHO–UNICEF principles on breastfeeding standards. This questionnaire consisted of six sections: social demographic factors, medical record around delivery, any previous breastfeeding or feeding experience, feeding intention in antenatal period, actual feeding practice after delivery, and breastfeeding support from the health system.

Childcare doctors in 36 community hospitals were trained as investigators by the experts prior to initiation of the survey. During visits from the mothers and children for routine childcare services, childcare doctors asked about the mothers’ willingness to participate in the survey, and a detailed survey was conducted after informed consent was obtained. The mothers completed the online questionnaire by scanning the QR code.

### 2.3. Breastfeeding Indicators

There are 17 IYCF indicators recommended by the 2021 edition of the WHO–UNICEF guidance [6]. According to the WHO definition, the six core breastfeeding indicators are ever breastfed (EvBF), early initiation of breastfeeding (EIBF), exclusively breastfeeding under six months (EBF), mixed milk feeding under six months (MixMF), continued breastfeeding 12–23 months (CBF), and infant-feeding area graphs (AGs). The breastfeeding indicators were calculated in compliance with the standard formulas provided and presented in forms of frequency/percentage.

### 2.4. Statistical Analysis

According to the aim of the survey, to estimate the exclusive breastfeeding rate under six months and the proportion of children aged 6–23 months who met the minimum acceptable dietary standards, the sampling population was divided into two groups. Systematic random sampling was used to select mothers at a particular interval from the Shanghai Child Care Management System. The sample sizes for the two age groups were calculated by using the following formula:

$$n=Z^{2}{}_{1-\alpha /2}\frac{1-P}{\epsilon^{2}P}$$

where *Z* is the Z score; *α*, the probability of a type I error; *ϵ*, sampling error; and *P*, the exclusive breastfeeding rate. According to the 2013 nutrition and health-monitoring data of Chinese residents from the Chinese Center for Disease Control and Prevention [11], the exclusive breastfeeding rate for the first six months in large cities in 2013 was 20.7%, and it is expected that the exclusive breastfeeding rate has increased in recent years, where the exclusive breastfeeding rate (P) was calculated as 25% and minimum acceptable diet (MAD) was calculated as 39.5% of the sample size. Thus, we aimed to survey at least 1358 children in the 0–5 months age group and 696 children in the 6–23 months to ensure adequate statistical power (adjusted for a nonresponse rate of 15%). In order to overcome the selection bias that occurs when only those participants who are interested in the survey respond to the questions, we sent gifts, such as storybooks for children to encourage participation.

We applied univariable and multivariable logistic regression models to identify the covariates associated with exclusive breastfeeding, the results of which are presented as crude odds ratios (ORs), adjusted ORs (^a^OR), and 95% confidence intervals (CIs). The *p*-values less than 0.05 in univariate analysis were included in the multivariate regression model. All data analyses were conducted using SAS 9.4 (SAS Institute, Inc., Cary, NC, USA).

## 3. Results

### 3.1. Demographic Characteristics

The general characteristics of the study population are given in Table 1. Over 15% of the children were born to women aged 35 years old or older; 84.8% of the mothers in our study have a bachelor’s degree or higher, and 70.2% are employed full time. Exposure to formula-milk marketing at different stages was also investigated; we found that self-reported exposure to marketing was pervasive during pregnancy (95.5%) and the postnatal stage (96.2%). The main channels for mothers’ exposure to formula-milk marketing at different stages are presented in Supplementary Table S1.

### 3.2. Breastfeeding Indicators

**Figure 1:**
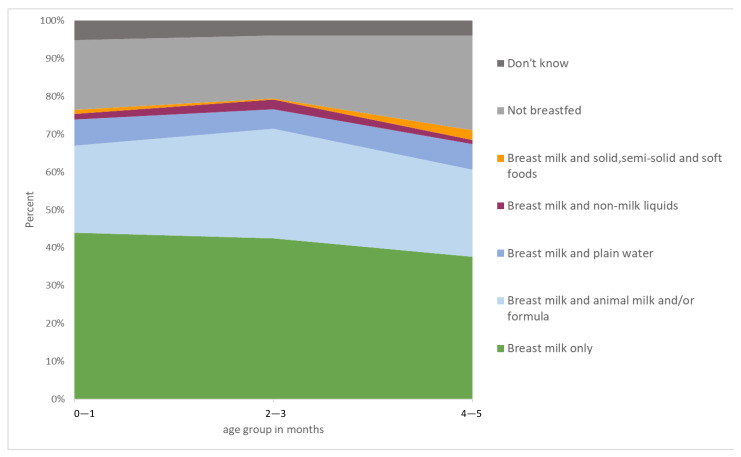
Area graph patterns of infant feeding practices under 6 months, by age group.

The breastfeeding indicators are presented in Table 2. Of 2665 infants under 24 months of age, 96.4% (2569/2665) were breastfed (infants reported to have been breastfed, regardless of the length of the period), and 60.3% had early initiation of breastfeeding. Among the 1677 infants aged 0–5 months, 727 were exclusively breastfed (EBF), 416 were mixed fed with formula and/or animal milk (MixBF), and 534 were not breastfed; the overall prevalence of EBF was 43.4% (727/1677). For infants aged 0–1 and 2–3 months, 43.2% and 47.3%, respectively, were exclusively breastfed. However, a sharp decline was seen among infants aged 4–5 months, with a rate of 37.6%, much lower than the rate of 45.5% for children aged 0–3 months. Figure 1 shows the patterns of infant-feeding practices by age group.

As for the newly added indicator, MixMF prevalence was 24.8%, which means that, of the 1677 infants aged 0–5 months, 416 were not fed exclusively with breast milk but with additional formula and/or animal milk during the previous day. The prevalence of continued breastfeeding among children aged 12–23 months (CBF) is 18.4%.

### 3.3. Determinants of Exclusive Breastfeeding

The results of univariate regression of the factors that influence EBF are summarized in Table 3. The univariate regression analysis indicates that a higher prevalence of exclusive breastfeeding was positively associated with determinants at multiple levels, including maternal education, household income, and breastfeeding intention at an individual level, and determinants related to the health systems, workplace, and employment at the settings level. EBF was more positively associated with higher maternal education than high-school education or lower; the higher the education level of the mothers, the more likely they were to choose EBF (OR = 2.00 and 95% CI: 1.48, 2.71 for bachelor’s degree; 2.56 and 95% CI: 1.74, 3.78 for master’s and above).

In the multivariate models shown in Table 4, we found that the EBF rate is significantly associated with women’s antenatal feeding intentions, belief in the health benefits of breast milk, and satisfaction with the support provided by the healthcare system. Compared with mothers who chose mixed milk feeding, women who chose exclusive breastfeeding were more likely to actually breastfeed their children in the first six months (^a^OR = 4.87; 95% CI: 3.46, 6.84). Regarding breastfeeding knowledge, such as the health benefits of breastfeeding in terms of a lower risk of obesity and diabetes, mothers who agree with the statements were more likely to exclusively breastfeed than mothers who had never heard them (^a^OR = 1.56; 95% CI: 1.08, 2.25). Moreover, professional health support was found to be another important determinant of breastfeeding promotion. Women being more satisfied with the support provided by the medical system after delivery was linked with a higher EBF rate (^a^OR = 2.56; 95% CI: 1.13, 5.80).

#### 3.3.1. Maternal Educational Level and Feeding Intentions in Pregnancy (or the Prenatal Period)

We calculated the detailed distribution of maternal educational levels in different feeding groups in children under six months (Table 5). Of 1663 mothers (14 of whom did not provide their education information), 84.8% had attained a bachelor’s degree or higher. Improved breastfeeding practices were observed in higher educational groups: in the subgroup of mothers with a master’s degree or doctorate, the proportions of exclusive breastfeeding, mixed feeding, and artificial feeding were 51.2%, 31.9%, and 16.9%, respectively, while in mothers with a high-school education or below, 29.1% exclusively breastfed, 21.6% chose mixed milk feeding, and 49.4% did not breastfeed.

In our study, 92.4% of the surveyed women expressed the desire to breastfeed in the prenatal period. Women’s infant feeding intentions during the prenatal period have a major effect on EBF (Supplementary Table S2). Among the 1036 mothers who planned on exclusive breastfeeding, more than half (58.1%) actually committed to EBF postpartum; among the 513 mothers who planned for mixed breastfeeding, only 100 (19.5%) actually committed to EBF in postpartum, while 239 (46.6%) employed mixed milk feeding and 174 (33.9%) did not breastfeed. All of the 16 mothers who had no intention to breastfeed during the prenatal stage were feeding their children with formula milk (15 artificial feeding and 1 mixed feeding). Mothers choose different feeding methods before delivery, and the reasons given are shown in Supplementary Table S3. Among the 1036 women who planned on exclusive breastfeeding, the top reason was that “breast milk is hygienic, convenient, and cost-effective” (82.3%). By contrast, of the 513 women who planned to feed their children with mixed milk, 402 lacked confidences in their breast milk being enough for their babies, and 82 thought that formula milk was as good as breast milk.

#### 3.3.2. Workplace and Employment

In our study, the female labor participation rate was 76%, with 70.2% in full-time employment and 5.8% in part-time employment. Among the 1274 working mothers, the top three occupation types were private enterprise (375, 29.4%), multinational corporation (240, 18.8%), and civil servant (231, 18.1%). In the univariate logistic analysis, the EBF rate was significantly associated with maternal employment status, type of employment, and length of paid maternal leave (Table 3). Compared with mothers employed full-time, mothers employed part-time had substantially lower rates of exclusive breastfeeding (OR = 0.45; 95% CI: 0.28, 0.72). When we compared the EBF rates between different occupational characteristics, we found that mothers in the civil service had the highest level of EBF (49.78%), followed by those employed by state-owned enterprises (48.77%) and multinational companies (43.75%). Regarding paid maternal leave, having a long maternity leave (≥120 days) was associated with increased odds of exclusive breastfeeding (OR = 1.35; 95% CI: 1.03, 1.77).

#### 3.3.3. Health Professional Support

Positive associations between health professional support and higher prevalence of exclusive breastfeeding were observed in our study. According to the univariate analysis, women who participated in a breastfeeding education class were informed about ways to solve breastfeeding problems and/or were satisfied with the support provided by medical staff after delivery were more likely to choose exclusive breastfeeding than women who did not attend the class or were less satisfied. Regarding the delivery mode and EIBF, vaginal birth and timely breastfeeding initiation were also factors positively associated with a higher rate of EBF.

#### 3.3.4. Marketing of Formula Milk and Exclusive Breastfeeding

Table 6 shows that, compared with the group of women who insisted on EBF, the proportion of women who believe that there is no association between breastfeeding and child intellectual development was higher in the MixMF and not-breastfed groups (11.3%, 8.8%, vs. 5.1%, *p* < 0.001). Nearly two-thirds of mothers who did not breastfeed had never heard about the advantages for children fed breast milk in terms of having a lower risk of obesity and diabetes, and this figure was much higher than the percentage in the EBF group (64.6% vs. 45.5%, *p* < 0.001).

**Figure 2:**
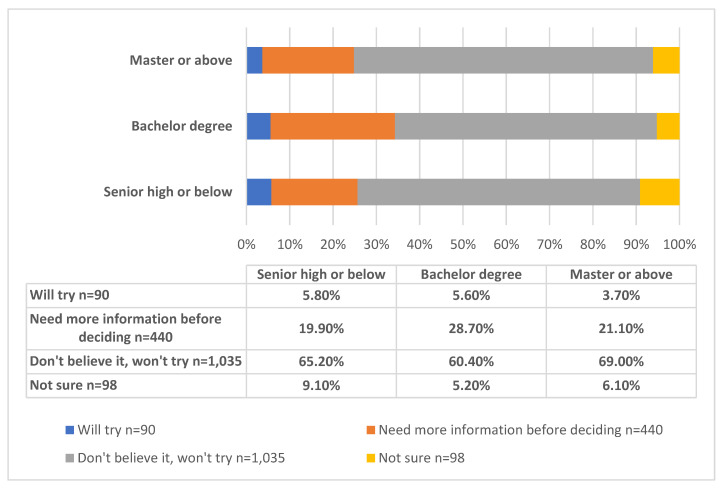
Response to the marketing massage that “formula milk can make children sleep well and reduce crying at night” among women with different educational backgrounds (N = 1663).

When they encountered an advertisement that stated that “Formula milk can make children sleep well and reduce crying at night”, 5.4% of the surveyed mothers chose to try it, while 62.2% did not believe this statement and said they would not try formula milk. With the increase in educational level, the proportion of mothers who believed the advertisement and tried the formula milk decreased. For example, only 3.8% of mothers with a high educational level (master’s or PhD) decided to try it, while 5.6% of mothers with a bachelor’s degree tried it, and 5.8% of mothers with a high-school education or below tried it based on this statement. Meanwhile, the proportion of those who answered “don’t believe the statement in ads and won’t try formula milk” increased with the improvement in the maternal education (Figure 2).

Not surprisingly, compared with mothers who insisted on exclusive breastfeeding, women who did not breastfeed were more willing to try a new formula advertised if told that it can help with children’s sleep (*p* < 0.001).

## 4. Discussion

### 4.1. Current Situation of Breastfeeding Practices in Shanghai

In this study, we assessed the breastfeeding practices of infants and young children in Shanghai, using standardized indicators, and we had high participant response rates and short recall periods. The rate of EBF in Shanghai recorded in our study (43.4%) was higher than that previously reported for the national level but still lower than the global target for 2025. The prevalence reached 96.4% for ever breastfeeding (Ever BF) and 60.3% for early initiation of breastfeeding (EIBF). The proportion of CBF was 18.4%, which is much lower than the global average (65.0%) [16]. Women being well prepared for infant- and young-child-feeding decisions in the prenatal period, being informed about breast milk, and being satisfied with the health support were favorable factors in terms of achieving optimal breastfeeding practices in Shanghai. The results of this study shed light on current breastfeeding practices and the priorities for breastfeeding-promotion intervention in Shanghai.

According to the conceptual model of the “Structural context for breastfeeding” proposed by Rollins et al., the current investigation includes determinants that operate at three levels [13]. Individual factors include maternal age, education, and breastfeeding intention; at the settings level, there are the effects of health systems, workplace, and employment; and at the structural level, there are social factors that affect the whole population, such as mothers’ exposure to formula advertisements at different stages.

### 4.2. Workplace and Employment

We noted that mothers engaged in informal work were less likely to breastfeed than those who were housewives or had full-time jobs. The result was consistent with the report by Duan et al., who found a lower prevalence of EBF among mothers who were not formally employed. This makes sense given that mothers working informally could be from the same disadvantaged population that lacks legislated social protection, such as paid maternity leave and income security. This finding is consistent with the national cross-sectional survey from 31 provinces in 2018. In their study, higher exclusive breastfeeding proportions were found among mothers being formally employed with ≥6 months of paid maternity leave (adjusted OR, 2.77; 95% CI, 1.65, 4.65) [12].

Subsequently, we further explored the breastfeeding practices of mothers according to different occupational types. The results indicate that women working in private enterprises are less likely to breastfeed than those in state civil positions (OR = 0.64; 95% CI: 0.46, 0.89). Our findings suggest that, in the presence of adequate maternity protection, employment is not necessarily a barrier to exclusive breastfeeding. It is important to note that, in our sample, 5.8% of the mothers worked informally, 24.0% were housewives, and the remaining 70.2% were formally employed. The proportion of working mothers in this study was much higher than the 16% reported by Tuan et al. in their survey of seven regions in China [13]. However, the female labor participation rates varied in different regions for reasons of economy, policy, history, and other factors [17].

Regarding the impact of the workplace and employment, our study indicates that Shanghai women having a paid maternal leave of more than 120 days was positively associated with exclusive breastfeeding, which supports the idea that maternity leave policies are effective at increasing exclusive breastfeeding [18]. Paid maternal leave was accordingly extended to 158 days in Shanghai in November 2021 [19]. According to the maternity protection legislation in Shanghai, the maternal-leave period exceeds the 18 weeks recommended by the International Labor Organization, thus indicating positive and supportive societal attitudes toward breastfeeding in Shanghai. The Chinese government and the Maternal and Child Health Committee have newly published a Breastfeeding Promotion Action Plan for 2021 to 2025, advocating a more flexible working schedule and a nursing break of at least one hour for lactating women.

### 4.3. Health Professional Support

The rate of women giving birth in a hospital was 100 percent in Shanghai. The adoption of breastfeeding practices is closely related to the health system, such as the policies of healthcare facilities and support from medical staff [20]. In response to the call for Baby-Friendly Hospital Initiatives (BFHIs) by WHO–UNICEF, 73 baby-friendly hospitals were launched across Shanghai prior to April 2022 [21]. The prevalence of EIBF was 60.3% in our study, which is similar to the rate recently reported by Huang et al. (67.7%) for one district hospital in Shanghai, which could be attributed to the routine practice of putting babies to their mothers’ breasts at baby-friendly hospitals [22]. A study conducted at eight baby-friendly hospitals with a total of 707 pregnant women in Shanghai found that better BF supportive services during hospitalization for childbirth were significantly associated with a higher rate of EBF at discharge and six months postpartum [23]. In our study, we also found that women who were more satisfied with the support offered by medical staff after delivery were more positive about exclusive breastfeeding. The results suggest that health professionals and governments should provide accurate, impartial breastfeeding information to parents.

In terms of birthing factors, the findings of the univariate analysis show that full-term delivery and vaginal birth are associated with higher exclusive breastfeeding rates. However, the associations were not significant in multivariate models.

### 4.4. Marketing of Formula Milk

Over 95% of surveyed women reported that they were exposed to formula-milk advertisements in the prenatal and/or postnatal stages. The percentage was similar to the self-reported exposure to formula-milk marketing in urban China (97%) reported by WHO and UNICEF in 2022. Formula-milk companies take advantage of parents’ anxieties and desires to influence their infant- and young-child-feeding decisions. The approach to formula-milk marketing and advertising in China is primarily attributable to a policy gap in legislation, monitoring, and enforcement. Despite women’s willingness to breastfeed, formula-marketing strategies can decrease their confidence in breastfeeding and in themselves [24].

Through univariate analysis, we found that women who agreed with the statement that breast milk is beneficial to children’s intellectual development or decreases the risk of obesity or diabetes were more likely to exclusively breastfeed. Our findings support widespread promotion of the known benefits of breastfeeding for women and children to counter the effects of formula-milk advertising. Based on previous data, the total milk-based-formula sales volume per infant/child in China grew by 106.0% from 2008 to 2013 [25]. Therefore, recognizing the urgency of countering the marketing message is of great importance; we should ensure that women are well informed with good knowledge and given guidance about breastfeeding [26]. Meanwhile, maternal confidence before the initiation of breastfeeding is of fundamental importance to the successful establishment and longer duration of breastfeeding. Our finding on the importance of maternal confidence in breastfeeding was similar to those reported in a previous review, which reported that the top reason for ceasing breastfeeding is “perceived breast milk insufficiency” [27]. It is critical that mothers are assisted in promoting and maintaining breastfeeding self-efficacy and to overcome their concerns based on commercially driven messages.

## 5. Conclusions

The rate of EBF under six months of age in Shanghai was higher than the corresponding rates of the most recent national surveys but still lower than the global target of 50% by 2025 [10,11,12,13]. Factors contributing to this higher rate are a result of several policy decisions resulting in more highly educated women in Shanghai, with access to health information and education, reporting a greater intention to breastfeed during pregnancy. Women also access health services in districts with a higher number of Baby-Friendly Hospital Initiatives (BFHIs), and women in formal employment have access to paid maternity leave. All of these factors have contributed to higher BF rates, showing that multiple strategies, from individual attributes and women’s employment conditions to breastfeeding knowledge and healthcare services, should be targeted to address the challenges in optimizing breastfeeding practices. In response, public health institutions, health professionals, and civil society should scale up investments to support women in achieving optimal breastfeeding practices.

## Figures and Tables

**Table 1:** Social, demographic, and birthing characteristics of the study’s mothers and children aged under 6 months (N = 1677).

Characteristics	N (%)
Maternal age at delivery	
20–24 y	110 (6.5)
25–29 y	578 (34.5)
30–34 y	694 (41.4)
≥35 y	295 (17.6)
Ethnicity	
Han	1636 (97.6)
Minorities	41 (2.4)
Marital status	
Married/co-habiting	1656 (98.8)
Other	21 (1.2)
Educational level	
Senior high or below	241 (14.4)
Bachelor’s degree	1209 (72.1)
Master’s or above	213 (12.7)
Don’t know/refuse to answer	14 (0.8)
Employment status	
Full-time employed	1177 (70.2)
Work informally/Part-time employed	97 (5.8)
Housewife	403 (24.0)
Type of employee	
State civil servant	231(18.1)
State-owned enterprise	203(15.9)
Multinational company	240(18.8)
Private enterprise	375 (29.4)
Self-employed	79 (6.2)
Others	146 (11.5)
Annual household income	
<150,000 CNY	682 (40.7)
150,000–300,000 CNY	514 (30.6)
>300,000 CNY	288 (17.2)
Don’t know/refuse to answer	193 (11.5)
Antenatal feeding intentions	
Exclusive breastfeeding	1036 (61.8)
Mixed milk feeding	513 (30.6)
Not breastfed	16 (0.9)
No plan	112 (6.7)
The first child	
Yes	1290 (76.9)
No	387(23.1)
Gender of child	
Boy	859 (51.2)
Girl	818 (48.8)
Delivery mode	
Vaginal birth	1066 (63.6)
Caesarean section	611 (36.4)
Gestational age	
Pre-term (28–36^+6^ gestational weeks)	96 (5.7)
Full-term (≥37 gestational weeks)	1581 (94.3)
Birth weight	
<2500 g	74 (4.4)
2500–3499 g	1507 (89.9)
≥4000 g	96 (5.7)
BMS marketing massage received *	
Pre-conception	474(28.3)
Pregnancy	1602(95.5)
Postnatal	1614(96.2)

* CNY, Chinese Yuan; BMS, breast-milk substitutes.

**Table 2:** Breastfeeding indicators of the study population (n = 2665).

	Indicators	Percentage (%)
1	Ever breastfed (EvBF)	96.4 (2569/2665)
2	Early initiation of breastfeeding (EIBF)	60.3 (1608/2665)
3	Exclusive breastfeeding under 6 months (EBF)	43.4 (727/1677)
	0–1 month	43.2 (235/544)
	2–3 months	47.3 (320/676)
	4–5 months	37.6 (172/457)
	0–3 months	45.5 (555/1220)
4	Mixed milk feeding under six months (MixMF)	24.8 (416/1677)
	0–1 month	28.7 (156/544)
	2–3 months	21.8 (147/676)
	4–5 months	24.7 (113/457)
5	Continued breastfeeding 12–23 months (CBF)	18.4 (100/543)
	12–15 months	25.3 (61/241)
	16–19 months	13.2 (26/197)
	20–23 months	12.4 (13/105)

**Table 3:** Determinants of exclusive breastfeeding (EBF) for children under 6 months in univariate analysis (n = 1677).

**Variable**	**Percentage of** **EBF**	**Univariable Model**	
		**OR (95% CI)**	***p*** **-Value**
Maternal age at delivery, years			0.546
≤24	40.91%	1.02 (0.66, 1.50)	
25–29	45.16%	1.22 (0.92, 1.62)	
30–34	43.52%	1.14 (0.86, 1.53)	
≥35	40.34%	**1.00**	
Educational level			**<0.001**
Master’s or above	51.17%	2.56 (1.74,3.78)	
Bachelor’s degree	45.08%	2.00 (1.48,2.71)	
Senior high or below	29.05%	**1.00**	
Employment status			**0.004**
Housewife	43.18%	0.94 (0.75, 1.18)	
Part-time employed	26.80%	**0.45 (0.28, 0.72)**	
Full-time employed	44.77%	1.00	
Type of employee			**0.047**
State civil servant	49.78%	**1.00**	
State-owned enterprise	48.77%	0.96 (0.66,1.40)	
Multinational company	43.75%	0.79 (0.55,1.13)	
Private enterprise	**38.73%**	**0.64 (0.46,0.89)**	
Self-employed	37.50%	0.61 (0.36,1.02)	
Others	40.27%	0.68 (0.45,1.03)	
Annual household income			**<0.001**
>300,000 CNY	50.00%	1.70 (1.28,2.24)	
150,000–300,000 CNY	47.28%	1.52 (1.21,1.92)	
<150,000 CNY	37.10%	**1.00**	
Gestational age			**0.005**
Full-term	44.21%	**1.92 (1.23, 3.02)**	
Pre-term	29.17%	1.00	
Delivery mode			**0.003**
Vaginal birth	46.06%	**1.36 (1.11, 1.66)**	
Caesarean section	38.63%	1.00	
Paid maternity leave			**0.029**
≥120 days	47.07%	1.35 (1.03,1.77)	
<119 days	39.74%	**1.00**	
Antenatal feeding intention			**<0.001**
Exclusive breastfeeding	57.05%	**5.70 (4.44, 7.32)**	
Mixed milk feeding	18.90%	1.00	
Viewpoint on the statement “Breast milk is beneficial to children intellectual development”			**<0.001**
Beneficial	38.94%	**1.62 (1.07, 2.45)**	
Beneficial with proper raising environment	48.67%	**2.41 (1.61, 3.60)**	
Not related	28.24%	1.00	
Viewpoint on the statement “Lower risk of obesity and diabetes for breast milk”			**<0.001**
Yes, I agree	55.31%	2.09 (1.65,2.65)	
Partly agree, Need further verification	45.03%	1.38 (1.09,1.76)	
I have never heard of it before	37.19%	1.00	
Participation in breastfeeding classes			**0.021**
Yes	45.78%	**1.26 (1.04, 1.53)**	
No	40.11%	1.00	
Overall satisfaction of information and support provided by healthcare system after delivery			**0.014**
Satisfied	46.12%	**1.98 (1.15, 3.43)**	
Not satisfied	30.16%	1.00	
Initiated early breastfeeding within an hour after birth			**<0.001**
Yes		**1.88 (1.54,2.30)**	
No		1.00	
Advertisement state that “formula milk can make children sleep well and reduce crying at night”			0.3250
Will try	36.56%	1.38 (0.89,2.14)	
Will gather more information before making decisions	42.65%	1.29 (0.82,2.03)	
Won’t try	44.33%	1.00	

**Table 4:** Determinants of exclusive breastfeeding (EBF) rate for children under 6 months in multivariate analysis (n = 1677).

	Adjusted OR (95% CI) *	*p*-Value
Antenatal feeding intention		<0.001
Exclusive breastfeeding	**4.87 (3.46, 6.84)**	
Mixed milk feeding	**1.00**	
Viewpoint on the statement “lower risk of obesity and diabetes for breast milk”		0.007
Yes, I agree	**1.56 (1.08,2.25)**	
Partly agree, Need further verification	1.31 (0.88,1.96)	
I have never heard of it before	**1.00**	
Overall satisfaction of information and support provided by healthcare system after delivery		0.030
Satisfied	**2.56 (1.13, 5.80)**	
Not satisfied	1.00	

* Adjusted model controlled for maternal educational level, employment status, type of employment annual household income, gestational age, delivery mode, paid maternal leave, antenatal feeding intention, breastfeeding knowledge, and significant factors in univariate analyses.

**Table 5:** Maternal educational level with children fed with different methods (N = 1663).

	EBF	MixMF	Not Breastfed	*p*-Value
	%	%	%	
Educational level	N1 = 724	N2 = 416	N3 = 534	**<0.0001**
Senior high or below	70 (29.1)	52 (21.6)	119 (49.4)	
Bachelor’s degree	545 (45.1)	295 (24.4)	369 (30.5)	
Master’s or above	109 (51.2)	68 (31.9)	36 (16.9)	

**Table 6:** Maternal attitude toward benefits of breastfeeding among mothers with different feeding practices.

	EBF	MixMF	Not Breastfed	*p*-Value
	%	%	%	
Attitude toward benefits of breast milk and formula				0.863
Different	600 (86.2)	345 (85.2)	412 (85.3)	
Almost the same	96 (13.8)	60 (14.8)	71 (14.7)	
Viewpoint of relationship between breastfeeding and children intellectual development				**<0.0001**
Beneficial	250 (34.4)	132 (31.7)	260 (48.7)	
Beneficial with proper raising environment	440 (60.5)	237 (57.0)	227 (42.5)	
Not related	37 (5.1)	47 (11.3)	47 (8.8)	
Viewpoint of lower risk of obesity and diabetes for breastfed children				**<0.0001**
I have never heard of it before	331 (45.5)	220 (52.9)	339 (64.5)	
Need further verification	172 (23.7)	101 (24.3)	109 (20.4)	
Yes, I agree	224 (30.8)	95 (22.8)	86 (16.1)	
Attitude toward “Formula milk can make children sleep well and reduce crying at night”				**<0.0001**
Will try	34 (5.0)	10 (2.5)	49 (9.9)	
Will gather more information before making decisions	186 (27.3)	123 (30.8)	133 (26.9)	
Won’t try	461 (67.7)	266 (66.7)	313 (63.2)	

## Data Availability

The questionnaire and datasets used during the current study are available from the corresponding author on reasonable request.

## Supplementary Materials

The following supporting information can be downloaded at https://www.mdpi.com/article/10.3390/nu14204429/s1. Supplementary Tables.
