# Supplementary material for: Breastfeeding Practices and Associated Factors in Shanghai: A Cross-Sectional Study
Source: Nutrients. 2022 Oct 21;14(20):4429. doi: 10.3390/nu14204429 (PMC9610171; doi:10.3390/nu14204429)
Supplement: Supplementary file 1 [file nutrients-14-04429-s001.zip › nutrients-1940619-supplementary.pdf]

Table S1. Multiple channels among mother exposure to formula milk marketing at different stage(N=1677)

	Promotions in supermarkets or maternity stores	Digital marketing. websites/WeChat/APPs, mass media, etc	Recommended by relatives and friends	Health professionals' recommendations	Other channel	Percentage of women exposed to formula milk marketing
**Pre-conception**	18.6%(312)	18.0%(301)	8.1%(135)	1.0%(17)	0.8%(13)	28.3%(474)
**Pregnancy**	57.7%(967)	52.1%(873)	30.1%(505)	5.2%(87)	11.5%(193)	95.5%(1,602)
**Postnatal**	58.6%(982)	55.0%(922)	31.3%(525)	7.2%(121)	10.0%(168)	96.2%(1,614)

Table S2. Women's infant feeding intentions during prenatal and different feeding practices (N=1677)

	EBF*	MixMF†	Artificial feeding
	n (%)	n (%)	n (%)
Antenatal feeding intention			
Exclusive breastfeeding	591(58.1)	158 (15.3)	287 (27.6)
Mixed milk feeding	100 (19.5)	239 (46.6)	174 (33.9)
Artificial feeding	0 (0.0)	1 (6.3)	15 (93.7)
No plan			

***EBF, exclusively breastfeeding; †MixMF, mixed milk feeding**

Table S3. Detailed Reasons for women's infant feeding intentions during prenatal (N=1677)

	N	Percentage(%)
**Reasons for intention of exclusive breastfeeding** (select all that apply)	1036	
**Exclusive breastfeeding is more hygienic, convenient, and economical**	853	82.3
**Exclusive breastfeeding is the best choice for children**	631	60.9
**Breastfeeding strengthens the mother-infant bond**	634	61.2
**Infant formula is bad for children's health**	127	12.3
**Other reasons**	9	0.9
**Reasons for intention of mixed milk feeding** (select all that apply)	513	
**Fear of breast milk insufficiency**	402	78.4
**Return to work**	205	40.0
**Formula milk is as good as breast milk**	82	16.0
**Poor health status**	45	8.8
**Friends/colleagues are mixed feeding their children**	112	21.8
**Other reasons**	15	2.9
**Reasons for intention of not breastfed** (select all that apply)	16	

**Poor health status**	2	12.5
**Return to work**	1	6.3
**Infant formula is as good as breast milk**	1	6.3
**Worries about their body or breasts will be out of shape due to breastfeeding**	3	18.8
**Miserable breastfeeding experience**	3	18.8
**Unwilling to breastfeed in public spaces**	1	6.3
**Other reasons**	7	43.8
**No Plan**	112	
